# Analysis of IL-17^+ ^cells in facet joints of patients with spondyloarthritis suggests that the innate immune pathway might be of greater relevance than the Th17-mediated adaptive immune response

**DOI:** 10.1186/ar3370

**Published:** 2011-06-20

**Authors:** Heiner Appel, René Maier, Peihua Wu, Rebecca Scheer, Axel Hempfing, Ralph Kayser, Andreas Thiel, Andreas Radbruch, Christoph Loddenkemper, Joachim Sieper

**Affiliations:** 1 Department of Gastroenterology, Infectiology and Rheumatology, Charité Berlin, Campus Benjamin Franklin, Hindenburgdamm 30, D-12200 Berlin, Germany; 2 Deutsches Rheumaforschungszentrum Berlin, Schumannstrassse 21/22, D-10117 Berlin, Germany; 3 Center for Spine Surgery, Werner-Wicker-Klinik, Im Kreuzfeld 4, D-34537 Bad Wildungen, Germany; 4 Department of Trauma and Reconstructive Surgery, Charité Berlin, Campus Benjamin Franklin, Hindenburgdamm 30, D-12200 Berlin, Germany; 5 Department of Pathology, Charité Berlin, Campus Benjamin Franklin, Hindenburgdamm 30, D-12200 Berlin, Germany

## Abstract

**Introduction:**

In this study, we analysed the number of IL-17^+ ^cells in facet joints, in the peripheral blood (PB) and synovial fluid (SF) of spondyloarthritis (SpA) patients and compared these results with those of patients with other rheumatic diseases and controls.

**Methods:**

Immunohistochemical analysis of IL-17^+ ^cells was performed in facet joints of 33 ankylosing spondylitis (AS) patients and compared with data from 20 osteoarthritis (OA) patients. The frequency of IL-17^+^CD4^+ ^T cells in PB and SF of SpA patients (PB *n *= 30, SF *n *= 11), rheumatoid arthritis (RA) patients (PB *n *= 14, SF *n *= 7), OA patients (PB *n *= 10) and healthy controls (PB *n *= 12) was analysed after stimulation with *Staphylococcus aureus *Enterotoxin B and phorbol 12-myristate 13-acetate/ionomycin and quantified by flow cytometry.

**Results:**

In AS facet joints, the frequency of IL-17-secreting cells was significantly higher than in samples obtained from OA patients (*P *< 0.001), with a slight predominance of IL-17^+ ^cells among the mononuclear cells (61.5% ± 14.9%) compared to cells with polysegmental nuclei. Immunofluorescence microscopy revealed that the majority of IL-17^+ ^cells were myeloperoxidase-positive (35.84 ± 13.06/high-power field (HPF) and CD15^+ ^neutrophils (24.25 ± 10.36/HPF), while CD3^+ ^T cells (0.51 ± 0.49/HPF) and AA-1^+ ^mast cells (2.28 ± 1.96/HPF) were less often IL-17-positive. The frequency of IL-17^+^CD4^+ ^T cells in the PB and SF of SpA patients did not differ significantly compared to RA patients, OA patients or healthy controls.

**Conclusions:**

Our data suggest an important role for IL-17 in the inflammatory processes in AS. However, the innate immune pathway might be of greater relevance than the Th17-mediated adaptive immune response.

## Introduction

Spondyloarthritis (SpA) comprises ankylosing spondylitis (AS), reactive arthritis, arthritis/spondylitis with inflammatory bowel disease and arthritis/spondylitis with psoriasis. Inflammatory back pain, a similar pattern of peripheral joint involvement with an asymmetrical arthritis predominantly of the lower limbs and the possible occurrence of sacroiliitis, spondylitis, enthesitis and uveitis are typical clinical features in this group of diseases [1]. SpA can be split into two categories, SpA with predominant axial involvement and SpA with predominant peripheral joint involvement, with both forms overlapping in about 20% to 30% of cases [1]. It has been suggested that SpA is a T-cell-driven disease [1]. *In vitro *analysis [2,3] and *in situ *analysis [4,5] have shown that both CD4^+ ^T cells and CD8^+ ^T cells might be involved in the pathogenesis of SpA.

CD4^+ ^effector T cells have been classified in the past as either Th1 cells predominantly secreting IFNγ or Th2 cells secreting IL-4. More recent work could define an IL-17-producing CD4^+ ^T-cell subtype termed Th17 cells [6,7]. The first evidence of a potential role of this cytokine was reported in human autoimmune diseases such as rheumatoid arthritis (RA) [8] and multiple sclerosis [9].

Numerous studies have been published recently investigating the frequency of Th17 cells in the peripheral blood (PB) of SpA patients in comparison to patients with other inflammatory joint diseases and controls. Contradictory results have been reported on the basis of either flow cytometry or ELISA [10-13].

In the present study, we investigated the presence and phenotype of IL-17^+ ^cells by analysing three different compartments in patients with SpA. Our results in PB and synovial fluid (SF) obtained from flow cytometric analysis were compared to results obtained from patients with RA (PB and SF) and controls (PB). Additionally, we performed an extensive analysis of IL-17^+ ^cells in bone specimens from AS patients. To our knowledge, this study is the first to describe *in situ *analysis of IL-17 expression in bone tissue samples.

## Materials and methods

### Patients

We obtained fresh PB from 30 patients with SpA, comprising 14 AS patients and 16 peripheral SpA patients, with the latter group comprising 10 patients with reactive arthritis, 3 with Crohn's disease and arthritis and 3 with undifferentiated peripheral SpA [14]. All AS patients fulfilled the modified New York criteria [15], and all peripheral SpA patients fulfilled the European Spondylarthropathy Study Group criteria [16]. We compared our data with those from 14 patients with RA [17], 10 patients with osteoarthritis (OA) and 12 healthy controls. We also analysed SF mononuclear cells (MNCs) from 11 SpA patients (3 patients with AS plus knee arthritis and 8 patients with peripheral SpA only) and from 7 patients with RA, with all samples taken from knee effusions. The patients' characteristics are shown in more detail in Table 1.

**Table 1:** Patient characteristics^a^

Characteristics	Rheumatoid arthritis		Spondyloarthritides	
	Synovial fluid	Peripheral blood	Synovial fluid	Peripheral blood
	(*n *= 7)	(*n *= 14)	(*n *= 11)	(*n *= 30)
Age, years	34.8 ± 12.4	53.4 ± 14.5	32.4 ± 11.0	35.8 ± 13.2
Disease duration, years	4.4 ± 7.6	2.6 ± 8.2	6.6 ± 5.2	6.09 ± 5.91
ESR, mm/hour	59.3 ± 13.8	52.2 ± 26.0	36.8 ± 20.2	27.7 ± 21.5
CRP, mg/mL	6.1 ± 4.9	5.4 ± 1.6	3.6 ± 2.7	2.3 ± 3.2
RF	6/7+	12/14+	4/4-	11/11-
Anti-CCP	4/7+	8/14+	3/3-	4/4-
HLA-B27	Not done	Not done	7/9+	21/26+
DAS28 score	5.6 ± 1.4	5.4 ± 1.8		
BASDAI score			2.3 ± 2.1	3.9 ± 2.6
DMARDs, *n*	All patients	12	3	None

^a^ESR = erythrocyte sedimentation rate; CRP = C-reactive protein; RF = rheumatoid factor; anti-CCP = anticyclic citrullinated peptide antibodies; HLA-B27 = human leucocyte antigen B27; DAS28 = disease activity score in 28 joints; BASDAI = Bath Ankylosing Spondylitis Disease Activity Index (measured in AS patients only); DMARDs = disease-modifying antirheumatic drugs. Data are expressed as means ± standard deviations unless otherwise indicated.

All samples from these patients and controls were freshly analysed. All patients and controls gave their informed consent to participate in the study. Permission to conduct this study was given by the local ethical committee of the Charité University Medicine Berlin, Campus Benjamin Franklin, Berlin, Germany.

Facet joints were obtained from 33 AS patients who had severe kyphosis with advanced ankylosis in the lumbar spine and had undergone surgery for polysegmental correction of rigid hyperkyphosis. Eighty-one percent of the AS patients were male, the mean age (± SD) of these patients was 48.6 ± 8.5 years, their mean disease duration was 23.6 ± 10.4 years, 73% of the patients were human leucocyte antigen B27-positive (HLA-B27^+^), 76% of AS patients were taking nonsteroidal antirheumatic drugs, four AS patients were taking non-TNF blocker disease-modifying drugs and one AS patient had discontinued adalimumab therapy four weeks before. Patients with spinal osteoporotic fractures and signs of diffuse idiopathic skeletal hyperostosis could be excluded on the basis of radiography [18]. Zygapophyseal joints were also obtained from patients with OA (*n *= 20) who had undergone surgery of the lumbar spine because of neurological deficits in the lower limbs caused by compression of the nerve roots. Seventy percent of the OA patients were female, and the mean age (± SD) of these patients was 71.4 ± 9.47 years. None of the patients had inflammatory diseases. All patients gave their informed consent to participate in this study.

### Tissue assessment

Tissue slices from zygapophyseal joints were prepared and examined as described before [5].

### Immunohistochemistry

Immunohistochemistry of paraffin-embedded zygapophyseal joints was performed to detect IL-17-expressing cells using a polyclonal anti-IL-17A antibody (R&D Systems, Wiesbaden-Nordenstadt, Germany). According to the manufacturer's description, this antibody has less than 1% cross-reactivity to human IL-17B, IL-17C, IL-17D and IL-17E and 10% cross-reactivity to IL-17F. Control experiments were performed with isotype controls and the anti-IL-17A antibody was also blocked by recombinant IL-17A (R&D Systems).

We used a rabbit anti-CD3 monoclonal antibody (clone SP-7; Thermo Scientific, Fremont, CA, USA) for double-staining of CD3^+ ^T cells, a mouse anti-CD20 monoclonal antibody (clone L26; Dako, Hamburg, Germany) for staining of CD20^+ ^B cells, an anti-CD15 antibody (clone MMA, Acris, Herfordt, Germany) for staining of CD15^+ ^neutrophils, an anti-mast cell tryptase monoclonal antibody (clone AA1, 1:400; Abcam, Cambridge, UK) for staining of mast cells, a rabbit anti-human myeloperoxidase (MPO) polyclonal antibody (Thermo Scientific) for staining of MPO^+ ^neutrophil precursors, an anti-CD56 monoclonal antibody (clone 1B6; Monosan, Uden, The Netherlands) for staining of natural killer (NK) cells, and the anti-glucocortin monoclonal antibody (clone Ret 40f; Dako, Glostrup, Denmark) for staining of erythrocyte precursors.

Quantification was performed as described before [5]. For this analysis, tissue sections with a detectable joint space were chosen. Areas in close proximity to these joint spaces were analysed.

### Staining for T-cell surface markers and intracellular cytokines and analysis by flow cytometry

T cells were stained after *in vitro *stimulation as described before [19]. Briefly, fresh MNCs were stimulated for six hours in the presence of 1 μg/mL anti-CD28 antibody alone (clone B27.2; Becton Dickinson, Heidelberg, Germany), 1 μg/mL *Staphylococcus aureus *Enterotoxin B (SEB) antibody (Sigma-Aldrich, Deisenhofen, Germany) and 25 ng/mL phorbol 12-myristate 13-acetate (PMA) (Sigma, Taufkirchen, Germany) plus 1 μg/mL ionomycin (Sigma, Taufkirchen, Germany). After two hours of stimulation, 10 μg/mL brefeldin A (Sigma Aldrich) was added to inhibit cytokine release from cells.

The following antibodies were used: anti-human CD4 (clone SK3; Becton Dickinson, anti-IL-17 (eBio64DEC17; eBiosciences, San Diego, CA, USA) and antihuman C-C chemokine receptor type 6 (CCR6) (clone 11A9; Becton Dickinson), as well as a mouse immunoglobulin G1 (IgG1) isotype control antibody (clone MOPC-1; Becton Dickinson). Positive cells were subsequently quantified by flow cytometry using a FACSCalibur flow cytometer with CellQuest software (Becton Dickinson, San Jose, CA, USA).

### CCR6 staining of IL-17^+ ^T cells

For staining of the cell surface marker CCR6 on IL-17^+ ^T cells, CD4^+ ^T cells were separated by magnetic absorbent cell sorting (MACS) as described before [3]. After *in vitro *stimulation with PMA/ionomycin as described above, CCR6^+ ^CD4^+ ^T cells were quantified by flow cytometry. As an isotype control, we used an antimouse IgG1 isotype antibody.

### Measurement of IL-17 secretion of CD4^+ ^T cells by ELISA

For measurement of IL-17 secretion by ELISA, CD4^+ ^T cells were also separated by MACS. After *in vitro *stimulation with PMA/ionomycin as described above, IL-17 was measured in the supernatant by IL-17 ELISA (Quantikine IL-17 ELISA; R&D Systems, Abington, UK) according to the manufacturer's instructions.

### Statistics

For data analysis, we used the Mann-Whitney *U *test or the Wilcoxon test if appropriate and SPSS for Windows software (SPSS, Inc., Chicago, IL, USA). Correlation analysis was performed by using the Pearson correlation coefficient. For the correlation analysis of ELISA and fluorescence-activated cell sorting analysis of IL-17^+ ^T cells, we calculated the nonparametric Spearman's rank correlation coefficient.

## Results

### *In situ *analysis of IL-17A^+ ^cells in facet joints of AS patients

**Figure 1:**
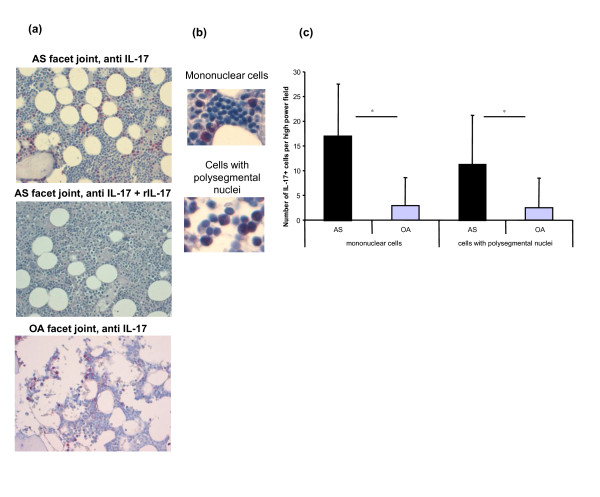
***In situ *analysis and quantification of IL-17^+ ^cells in patients with ankylosing spondylitis or osteoarthritis**. **(a) ***In situ *analysis of IL-17^+ ^cells in facet joints of ankylosing spondylitis (AS) (top) and osteoarthritis (OA) (bottom) patients. The specificity of IL-17 staining (top) is shown by blocking the anti-IL-17 antibody with recombinant IL-17 (rIL-17) (middle). **(b) **and **(c) **The frequency of IL-17-secreting mononuclear cells and cells with polysegmental nuclei in the bone marrow of AS facet joints was significantly higher in AS than in OA facet joints. *P < 0.001.

Light microscopic analysis revealed that not only MNCs but also mature neutrophils with polysegmented nuclei (PNCs) were IL-17A^+ ^(Figure 1a, top: AS facet joint; 1a bottom: OA facet joint). The specificity of IL-17 staining could be underlined by blocking the anti-IL-17 antibody and the positive staining with recombinant IL-17 in each experiment (Figure 1a, middle). The frequency of IL-17^+ ^MNCs (Figure 1b, top) and IL-17^+ ^PNCs (Figure 1b, bottom) was significantly higher in the bone marrow of AS facet joints (mean MNCs ± SD 17.08 ± 10.41/high-power field (HPF), PNCs 11.78 ± 9.92/HPF) compared to OA facet joints (MNCs 2.9 ± 5.67/HPF, *P *< 0.001; PNCs 2.55 ± 5.97/HPF, *P *< 0.001) (Figure 1c). Of all IL-17^+ ^cells, 61.5% ± 14.9% were MNCs in AS patients and 57.0% ± 11.4% were MNCs in OA patients (*P *> 0.05). There was a positive correlation between IL-17^+ ^MNCs and IL-17^+ ^PNCs in both AS patients (*r *= 0.634, *P *< 0.001) and OA patients (*r *= 0.991, *P *< 0.001).

**Figure 2:**
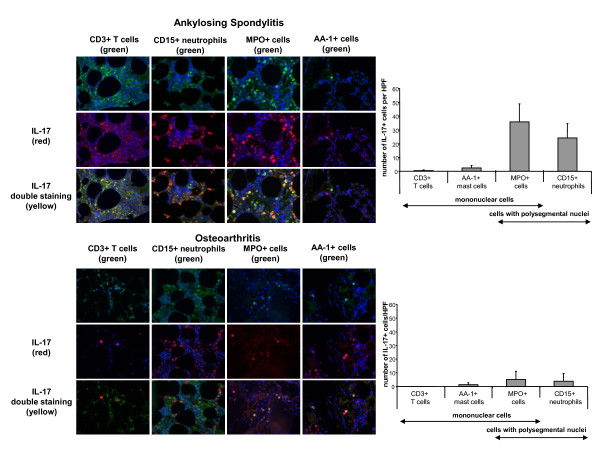
***In situ *immunofluorescence analysis of IL-17^+ ^cells**. *In situ *analysis of IL-17^+ ^cells in facet joints of ankylosing spondylitis (AS) patients and patients with osteoarthritis (OA) by using immunofluorescence microscopy. Double-staining reveals that myeloperoxidase-positive (MPO^+^) and CD15^+ ^cells are the major source of IL-17 expression. The frequency of these cells was significantly higher in AS than in OA (*P *< 0.05 in both cases). The population of MPO^+ ^cells included mononuclear cells and cells with polysegmental nuclei. Th17 cells and mast cells are also a source for IL-17^+ ^expression, both of which were significantly higher in AS patients than in OA patients (*P *< 0.05 in both cases).

Subsequently, we identified the IL-17-producing cell type in more detail in a subgroup of 12 AS patients and 10 OA patients by double-staining and immunofluorescence microscopy. By using this method, we could determine that neutrophil precursors detected by MPO staining (35.84 ± 13.04/HPF) and CD15^+ ^neutrophils (24.25 ± 10.36/HPF) were by far the most frequent cell populations expressing IL-17. Only a small proportion of AA-1^+ ^mast cells (2.28 ± 1.16/HPF) and an even smaller proportion of CD3^+ ^T cells (0.51 ± 0.49/HPF) were IL-17^+ ^(Figure 2). In OA patients, the frequency of all IL-17^+ ^cell types was significantly lower (*P *< 0.05 in all cases), although there was a similar percentage of the different IL-17^+ ^cell types: CD3^+ ^T cells 0.1 ± 0.1/HPF, AA-1^+ ^mast cells 1.36 ± 1.53/HPF, neutrophil precursors detected by MPO staining 5.04 ± 6.15/HPF and CD15^+ ^neutrophils 3.88 ± 5.75/HPF (Figure 2). Within the population of MPO^+ ^cells, 63.5% and 36.5% of cells were MNCs and PNCs in AS patients, respectively, and 65.1% and 34.9% of cells were MNCs and PNCs in OA patients, respectively.

Double-staining with an anti-CD20 antibody directed against B cells, with an anti-CD56 antibody directed against natural killer cells and with an antibody against erythrocyte precursors did not reveal IL-17^+ ^cells among these cell types (data not shown).

In some of the AS patients and OA patients, age-matched analysis was possible. In these patients, the higher frequency of IL-17^+ ^cells in AS patients compared to OA patients could be confirmed. In the three 55-, 63-and 67-year-old OA patients, 0, 0 and 0.7 IL-17^+ ^cells/HPF were found, respectively, compared to the three 57-, 63-and 67-year-old AS patients who had 21.1, 21.0 and 1.7 cells/HPF, respectively.

### Similar frequency of CD4^+^IL-17^+ ^cells in the peripheral blood of SpA patients compared to RA patients, OA patients and healthy controls

**Figure 3:**
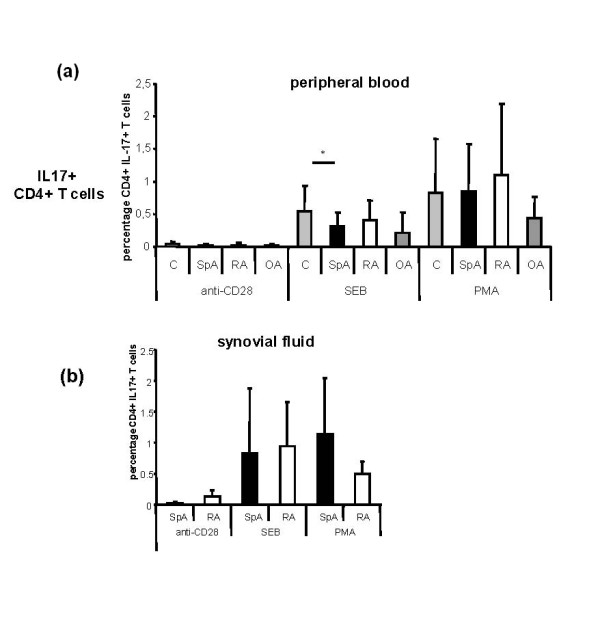
**Peripheral and synovial CD4^+^IL-17^+ ^T cell levels in spondyloarthritis, rheumatoid arthritis and controls**. Analysis of **(a) **peripheral blood (PB) and **(b) **synovial fluid (SF) CD4^+^IL-17^+ ^T cells in spondyloarthritis (SpA) patients (PB *n *= 30, SF *n *= 11), rheumatoid arthritis (RA) patients (PB *n *= 14, SF *n *= 7), osteoarthritis (OA) patients (PB *n *= 10) and healthy controls (C) (PB *n *= 12). Similar levels of PB and SF CD4^+^IL-17^+ ^T cells after stimulation with phorbol 12-myristate 13-acetate (PMA)/ionomycin or *Staphylococcus aureus *Enterotoxin B (SEB) antibodies are seen when SpA patients are compared to RA patients. The frequency of PB CD4^+^IL-17^+ ^T cells was only significantly lower in SpA patients than in controls when stimulated with SEB antibodies (*P *< 0.05).

We observed similar levels of CD4^+^IL-17^+ ^PB T cells (Figure 3a) after stimulation with PMA/ionomycin or SEB in SpA patients compared to RA patients, OA patients and healthy controls. Except for SEB stimulation (AS in comparison to controls, *P *< 0.05), we did not observe significant differences (Figure 3a). The lowest frequency of CD4^+^IL-17^+ ^T cells was observed in OA patients.

### Similar frequency of CD4^+^IL-17A^+ ^cells in the synovial fluid of SpA and RA patients

The frequency of SF CD4^+ ^IL-17^+ ^cells (Figure 3b) did not differ significantly when SpA and RA patients were compared.

### Higher frequency of CD4^+^IL-17A^+ ^cells in the synovial fluid in comparison to peripheral blood of SpA and RA patients

We further addressed the question whether there was a different frequency of IL-17^+ ^T cells in PB or SF. The frequency of CD4^+^IL-17^+ ^T cells was higher in SF than in PB in SpA and RA patients. However, except for SEB stimulation of CD4^+ ^T cells in RA patients (*P *= 0.041), this finding was not statistically significant. This was also seen in a subgroup of five AS patients with matched SF and PB samples. The mean PB percentages (± SD) of CD4^+^IL-17^+ ^cells were 0.02% ± 0.01% after stimulation with anti-CD28, 0.19% ± 0.05% after stimulation with SEB and 0.67% ± 0.61% after stimulation with PMA/ionomycin (all *P *> 0.05). The mean SF percentages (± SD) of CD4^+^IL-17^+ ^cells were 0.02% ± 0.01% after stimulation with anti-CD28, 0.71% ± 1.24% after stimulation with SEB and 0.92% ± 0.48% after stimulation with PMA/ionomycin (all *P *> 0.05)

### CCR6 expression in IL-17^+ ^T cells

**Figure 4:**
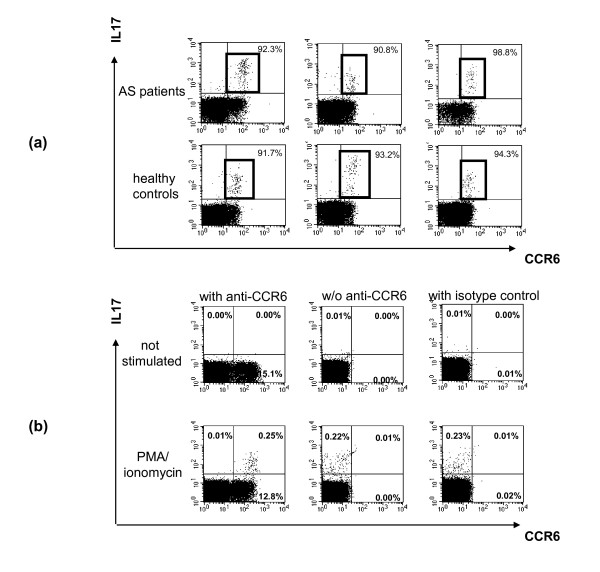
**CCR6 expression in CD4^+^IL-17^+ ^T cells**. **(a) **In three ankylosing spondylitis (AS) patients and three healthy controls, the expression of C-C chemokine receptor type 6 (CCR6) in CD4^+^IL-17^+ ^T cells derived from peripheral blood was analysed after *in vitro *stimulation with phorbol 12-myristate 13-acetate (PMA)/ionomycin antibodies. Percentages indicate the relative number of CCR6^+ ^cells to the total number of CD4^+^IL-17^+ ^T cells (top row of dot blot analysis). **(b) **Using an isotype control antibody after T-cell stimulation with PMA/ionomycin (bottom right) antibodies revealed no positive staining, confirming the specificity of CCR6 staining (bottom left). Without such T-cell stimulation, no CCR6^+^IL-17^+ ^T cells were detected.

Because Th17 cells express CCR6 nearly exclusively on their surface [19], we analysed the expression of CCR6 on the cell surface of peripheral CD4^+^IL-17^+ ^T cells, after MACS separation of CD4^+ ^T cells and *in vitro *stimulation, obtained from three AS patients and three healthy controls. The results are shown in Figure 4a. More than 90% of IL-17-secreting CD4^+ ^T cells were also CCR6^+ ^in all cases, supporting the specificity of our Th17 staining. Use of an isotype control antibody revealed negative staining, confirming the specificity of the staining. CD4^+^IL-17^+ ^CCR6^+ ^T cells were not detected without PMA/ionomycin stimulation (Figure 4b).

### IL-17 secretion by T cells measured by ELISA

The specificity of Th17 staining by flow cytometry was further confirmed when CD4^+ ^T cells derived from the PB from three AS patients and three healthy controls were separated by MACS and IL-17 secretion was measured in the supernatant by ELISA after *in vitro *stimulation. When IL-17 secretion after *in vitro *stimulation was compared with the intracellular cytokine staining data for CD4^+ ^T cells from the same patients, a good correlation of *r *= 0.66 was found, further confirming the specificity of the IL-17 staining (Table 2).

**Table 2:** IL-17 in CD4^+ ^T cells: Comparison of ELISA and intracellular cytokine staining^a^

Individuals	ELISA for IL-17-secreting CD4^+ ^T cells		Intracellular cytokine staining for CD4^+^IL-17^+ ^T cells	
	Without stimulation	With PMA/ionomycin	Without stimulation	With PMA/ionomycin
AS patient 1	0 pg/nL	1,667.9 pg/nL	0.0% of CD4^+ ^T cells	0.98% of CD4^+ ^T cells
AS patient 2	0 pg/nL	967.8 pg/nL	0.0% of CD4^+ ^T cells	0.47% of CD4^+ ^T cells
AS patient 3	0 pg/nL	1,398.5 pg/nL	0.0% of CD4^+ ^T cells	1.72% of CD4^+ ^T cells
Control 1	0 pg/nL	1,784.4 pg/nL	0.0% of CD4^+ ^T cells	1.21% of CD4^+ ^T cells
Control 2	0 pg/nL	277.9 pg/nL	0.0% of CD4^+ ^T cells	0.57% of CD4^+ ^T cells
Control 3	0 pg/nL	920.5 pg/nL	0.0% of CD4^+ ^T cells	0.69% of CD4^+ ^T cells

^a^PMA = phorbol 12-myristate 13-acetate; AS = ankylosing spondylitis.

## Discussion

In this study, we analysed the frequency of IL-17^+ ^cells in three different compartments of patients with spondyloarthritides. The most prominent finding was a significantly higher number of IL-17^+ ^cells at the primary site of inflammation in the subchondral bone marrow of affected facet joints [5] in AS patients compared to OA patients. Facet joints from patients with other inflammatory rheumatic diseases, such as RA patients, would have been of interest for comparison in this analysis, but such surgical procedures are rarely performed in RA patients. Interestingly, IL-17^+ ^cells were almost similarly distributed among the MNC and PNC populations, with a slight predominance in the PNC population. Surprisingly, immunofluorescence double-staining *in situ *showed that the clear majority of the IL-17^+ ^cells were found among the CD15^+ ^neutrophils (24.25 ± 10.36/HPF) and among the MPO^+ ^cells of the myeloid lineage (35.84 ± 13.04/HPF), while CD3^+ ^T cells (0.51 ± 0.49/HPF) and mast cells (2.28 ± 1.16/HPF) constituted only a small proportion of IL-17^+ ^cells. Staining for other cell types (B cells, NK cells and erythrocyte precursors) could exclude these cells as other sources of IL-17. However, we cannot exclude that, in the early phase of the disease, such a finding might be different because our current results were obtained in patients with advanced AS.

These data suggest that IL-17^+^-secreting cells other than the Th17 cells are of relevance in local inflammation in AS. Investigators in two recent studies on synovial membranes from patients with RA [20] or peripheral SpA, including psoriatic arthritis (PsA) [21], also showed that IL-17-producing cells other than Th17 cells are of relevance. In both RA and PsA patients, mast cells were the major source of IL-17, while Th17 cells were rather rare among the IL-17-producing cells, similar to the findings in our study.

There have previously been some indirect hints that Th17 cells might play a role in the pathogenesis of SpA. An extensive genotype analysis performed recently revealed that AS is closely linked to polymorphisms in the IL-23 receptor gene [22], suggesting that Th17 might be of relevance, although the functional consequence of this IL-23 polymorphism has not been clarified. Furthermore, in HLA-B27/human β_2_-microglobulin-transgenic rats, a possible animal model of SpA, HLA-B27 misfolding and the unfolded protein response resulted in a strongly activated IL-23/IL-17 axis in the colon of B27/β_2_-microglobulin-transgenic rats with SpA-like disease [23].

Nonetheless, our results and the studies of RA patients [20] and peripheral SpA patients [21] indicate that T cells might have been overestimated as the source of IL-17 in these chronic inflammatory diseases and that an innate immune response in the context of IL-17 might be of relevance. Interestingly, a high frequency of IL-17^+ ^mast cells and IL-17^+ ^neutrophils, as well as a low frequency of Th17 cells, was also described in the biopsies of skin lesions of psoriasis patients [24]. An analysis of patients with ulcerative colitis revealed an elevated number of Th17 cells located in the lamina propria of inflammatory lesions [25], but the relative number of Th17 cells in comparison to other IL-17^+ ^cells was not analysed. On the basis of the results of our investigation, however, we cannot exclude the possibility that Th17 cells are of any relevance in AS. Although the frequency was relatively low, it was higher than in the control group and might be sufficient to orchestrate an immune response.

In our study, mast cells as a source of IL-17 were much less frequent than in RA patients [20] and psoriasis patients [21], and also compared to neutrophils and their precursors. The IL-17 receptor A is highly expressed in hematopoietic cells [26]. Whether the positive staining of neutrophils and MPO^+ ^precursor cells is due to autocrine secretion or is caused by binding of IL-17 at the IL-17 receptor could not be determined by our staining. Interestingly, it has been well described that T-cell-derived IL-17 is an important growth factor for granulopoiesis in humans [27]. Although the involvement of IL-17^+ ^neutrophils in inflammatory processes has also been reported [26,28,29], though not yet in patients with SpA, further confirmation that IL-17 is produced by neutrophils by other methods, such as by *in situ *hybridisation, would be warranted. Very recently, Li *et al*. [30] presented impressive data showing that IL-17-producing neutrophils participated in innate immune responses in a mouse model of kidney reperfusion injury. Staining for MPO, which is produced during myeloid differentiation in the bone marrow by neutrophils and their precursors [31], is rather specific for this cell lineage, and these precursor cells appear in the shape of MNCs. Nonetheless, a more detailed characterisation of the MPO^+ ^precursor cells found in our present study will be of interest in the future.

Because AS patients are considerably younger than OA patients, an age-matched subanalysis was possible in only three AS patients and three OA patients, confirming a clearly higher number of IL-17^+ ^cells in AS patients than in OA patients. Nonetheless, the lack of a larger age-matched control group, which will also not easily be found in possible follow-up investigations, can be seen as a limitation of our study.

In PB and SF of SpA patients, we only looked for the frequency of Th17 cells and not for other IL-17-producing cells. Here we observed no significant differences compared to RA patients, OA patients or healthy controls with regard to PB and compared to RA with regard to SF. These results confirm our immunohistological analysis of the bone that Th17 cells do not seem to play an important role in AS. Previous investigations reported a higher number of Th17 cells in the PB of SpA patients [10,11], while reduced levels of IL-17 were found in the SF of SpA patients in another study. Our data also indicate that the analysis of IL-17-producing cells in inflamed tissue might be more informative than in PB or SF.

It is currently unclear whether the overexpression of IL-17 in inflammatory lesions of different autoimmune diseases indicates an extraordinary pathogenic role of this cytokine during inflammatory processes or whether this is just a secondary reaction [32]. However, the good clinical response in trials with the anti-IL-17 antibody secukinumab in RA patients [33], psoriasis patients and AS patients [34] suggests that IL-17 might indeed play a role in these diseases. The observed rather rapid clinical response in these trials might be a further hint that direct inhibition of soluble IL-17 is more important than modulation of a T-cell response. Interestingly, a similar discussion of the effect of TNF blockers, mainly neutralisation of soluble TNF or modulation of T-cell responses, has been ongoing over the past few years, but the issue has not been resolved yet [35].

## Conclusions

Our study suggest an important role for IL-17 during inflammatory processes in SpA patients. Our data also indicate that the innate immune pathway, mostly mediated through neutrophils and less via mast cells, might play a relevant role during inflammatory processes in AS patients, while Th17 cells seem to be of less importance.

## Abbreviations

AS: ankylosing spondylitis; ELISA: enzyme-linked immunosorbent assay; IL: interleukin; IFN: interferon; MACS: magnetic absorbent cell sorting; MNC: mononuclear cell; MPO: myeloperoxidase; OA: osteoarthritis; PB: peripheral blood; PNCs: cells with polysegmental nuclei; RA: rheumatoid arthritis; SF: synovial fluid; SpA: spondyloarthritis; TNFα: tumour necrosis factor α.

## Competing interests

The authors declare that they have no competing interests.

## Authors' contributions

HA and JS designed the study, analysed the data and drafted the manuscript. RH, PW and RS participated in the data collection, performed the data analysis and helped in the drafting of the manuscript. AH and RK participated in the data collection and helped in the drafting of the manuscript. AT and AR analysed the data and participated in the drafting of the manuscript. All authors were contributed to discussions and read and approved the final manuscript.
